# The Integrator Complex Subunit 6 (Ints6) Confines the Dorsal Organizer in Vertebrate Embryogenesis

**DOI:** 10.1371/journal.pgen.1003822

**Published:** 2013-10-31

**Authors:** Lee D. Kapp, Elliott W. Abrams, Florence L. Marlow, Mary C. Mullins

**Affiliations:** Perelman School of Medicine at the University of Pennsylvania, Department of Cell and Developmental Biology, Philadelphia, Pennsylvania, United States of America; Stanford University School of Medicine, United States of America

## Abstract

Dorsoventral patterning of the embryonic axis relies upon the mutual antagonism of competing signaling pathways to establish a balance between ventralizing BMP signaling and dorsal cell fate specification mediated by the organizer. In zebrafish, the initial embryo-wide domain of BMP signaling is refined into a morphogenetic gradient following activation dorsally of a maternal Wnt pathway. The accumulation of β-catenin in nuclei on the dorsal side of the embryo then leads to repression of BMP signaling dorsally and the induction of dorsal cell fates mediated by Nodal and FGF signaling. A separate Wnt pathway operates zygotically via Wnt8a to limit dorsal cell fate specification and maintain the expression of ventralizing genes in ventrolateral domains. We have isolated a recessive dorsalizing maternal-effect mutation disrupting the gene encoding Integrator Complex Subunit 6 (Ints6). Due to widespread de-repression of dorsal organizer genes, embryos from mutant mothers fail to maintain expression of BMP ligands, fail to fully express *vox* and *ved*, two mediators of Wnt8a, display delayed cell movements during gastrulation, and severe dorsalization. Consistent with radial dorsalization, affected embryos display multiple independent axial domains along with ectopic dorsal forerunner cells. Limiting Nodal signaling or restoring BMP signaling restores wild-type patterning to affected embryos. Our results are consistent with a novel role for Ints6 in restricting the vertebrate organizer to a dorsal domain in embryonic patterning.

## Author Summary

A complex integration of signaling pathways establishes the body plan of the vertebrate embryo. The dorsal side of the embryo is defined by the organizer, a specialized field of cells that breaks the symmetry of the zebrafish blastula by instructing neighboring cells to adopt dorsal fates based on their proximity. The isolation of mutant genes in the zebrafish has identified many genes required for embryonic development. However, our knowledge of the molecular mechanisms integrating different signaling pathways within a gene regulatory network to properly pattern the embryo is still incomplete. We isolated a recessive maternal effect mutation in the *integrator complex subunit 6* (*ints6*) gene that leads to a circumferential expansion of the organizer and the formation of dorsal tissues at the expense of ventral tissues. Currently, the only reported role for the Integrator Complex is to mediate processing of snRNAs of the spliceosome. Our molecular genetic approach indicates that *ints6* confines the organizer to dorsal domains, preventing it from extending around the margin into ventral domains. Thus, we have determined a novel role for a highly conserved component of an RNA processing machine.

## Introduction

The vertebrate embryonic dorsal organizer, historically referred to as the Spemann organizer, breaks the symmetry of the blastula by defining its dorsal side and ultimately gives rise to axial mesoderm, which forms the notochord, the defining anatomical feature of the chordate lineage. In fish and frogs, induction of the organizer relies on a maternal Wnt signaling pathway that leads to the accumulation of β-catenin in nuclei on the prospective dorsal side of the embryo [1], [2]. A primary function of the organizer is to induce a region in the embryo that is competent to adopt dorsal fates, such as prechordal plate mesoderm and neural ectoderm, in the presence of widespread ventralizing BMP signaling.

Proper partitioning of axial versus non-axial cell fates during gastrulation is essential to ensure proper embryonic patterning. BMP signaling patterns tissues along the dorsoventral axis (DV), but does not act to partition axial versus non-axial fates. For example, in zebrafish *bmp2b* (*swirl*) ligand mutant embryos, loss of BMP signaling causes the expansion of dorsal neurectodermal and non-axial dorsal mesodermal cell fates at the expense of ventral cell fates without expanding the organizer itself [3], [4], [5], [6]. Thus, in the absence of ventral cell fate specification, other mechanisms ensure that the organizer is confined dorsally.

In zebrafish and Xenopus, several maternal and zygotic genes function to restrict the organizer to dorsal regions. Three related homeodomain-containing transcriptional repressors, Vox, Vent, and Ved play a key role in repressing dorsal organizer gene expression ventrolaterally in zebrafish [7], [8], [9], [10], [11]. These repressors are expressed ventrally and dorsolaterally, and their deficiency causes dorsal organizer gene expression to expand around the ventrolateral margin during late blastula stages at the expense of ventrolateral tissues. *Xenopus* Vox and Vent have been shown to directly repress the expression of the organizer genes *chordin* (*chd*) and *goosecoid* (*gsc*) [12], [13] and depletion of Gsc has been reported to lead to a 25-fold increase in *vent* expression [14]. Similarly in zebrafish, Vox and Vent have been shown to bind to the *gsc* promoter and to physically associate with Gsc protein [8], [9]. These and other data [15], [16] illustrate the cross-regulatory interactions between opposing ventralizing and dorsalizing transcriptional repressors that are essential for proper embryonic patterning.

Several additional genes are known to restrict organizer gene expression to dorsal regions and modulate the expression of *vox*, *vent*, and *ved*. Knockdown of Runx2bt2, a maternal isoform of Runx2b, delays induction of *vox* and *vent*, and eliminates *ved* expression [17]. Embryos deficient in maternal Runx2bt2 exhibit an expansion of dorsal organizer gene expression at late blastula stages with a reciprocal loss of ventrolateral tissues [17]. Expression of *vox*, *vent*, and *ved* is maintained during late blastula and early gastrula stages by zygotic Wnt8a signaling [10], [18]. By mid gastrulation, expression of these ventralizing transcriptional repressors is maintained by BMP signaling [10], [19]. Thus, a gene regulatory network involving Runx2bt2, Wnt8a and BMP signaling converges on *vox*, *vent*, and *ved* to maintain the specification of non-axial mesoderm.

The maternally supplied transcription factor *pou5f3* (previously called *pou5f1*) also functions in restricting the organizer to the dorsal midline. Maternal-zygotic deficiency of *pou5f3* (MZ*pou5f3*) leads to severe dorsalization resulting from derepression of organizer genes ventrolaterally in the embryonic margin, and incomplete induction of the BMP pathway [20]. MZ*pou5f3* mutants also exhibit aberrant morphogenesis and fail to form endoderm [20], [21], [22]. Pou5f3 likely functions as a transcriptional activator of genes, including *vox*, that are required to repress dorsal organizer gene expression ventrolaterally [23], [24]. Thus, Pou5f3 is another mediator of organizer gene repression operating in parallel to the Wnt8a pathway and partially through the BMP pathway.

One of the earliest organizer genes induced downstream of the maternal Wnt pathway in zebrafish is *bozozok* (*boz*), a direct transcriptional repressor of *bmp2b*, *vox*, *vent*, and *ved* expression [8], [25], [26], [27], [28], [29], [30]. *boz* mutant embryos fail to form prechordal plate, notochord, forebrain, and ventral neural structures and display an increase of ventroposterior mesoderm [25]. *boz* mutant embryos can be rescued by suppressing Wnt8a signaling, indicating that Boz antagonizes zygotic *wnt8a* expression in the organizer to block non-axial fate development in the dorsal embryonic midline and allow axial development [26]. Boz stability is modulated by Lnx2b, a maternally supplied E3 ubiquitin ligase that can directly bind and ubiquitinate Boz [31], [32]. Loss of Lnx2b causes expression of *boz* and other organizer genes to expand into lateral regions of the late blastula, illustrating the importance of proper turnover of Boz.

The transcriptional repressors Vox, Vent, and Ved are essential for partitioning the mesoderm into axial versus non-axial domains in response to positive regulation from Runx2bt2, Pou5f3, the Wnt8, and BMP pathways, and negative inputs from dorsalizing transcriptional repressors such as Boz and Gsc. It is less clear how these pathways are molecularly integrated to regulate *vox*, *vent*, and *ved* expression and it is likely that additional maternally-provided factors function in this process. Accordingly, we performed a genetic screen for maternal-effect mutations to identify novel mediators of vertebrate embryonic patterning.

We isolated a novel recessive maternal-effect mutation *p18ahub* that causes a profound reduction in ventrolateral mesoderm with a reciprocal expansion in axial mesoderm, and frequently multiple independent axial-like domains. Consistent with radial expansion of the organizer, *p18ahub* mutant females produce embryos exhibiting ectopic dorsal forerunner cells, a unique population of non-involuting mesendodermal cells at the dorsal margin [33], [34], [35]. We can rescue *p18ahub* dorsalized mutant embryos either by limiting Nodal signaling or restoring BMP signaling. We determined through positional cloning that *p18ahub* is a mutation disrupting the *integrator complex subunit 6 (ints6)* gene, which encodes a highly conserved component of the Integrator Complex, a large multisubunit complex implicated in 3′ end processing of spliceosomal snRNAs [36]. Previously, *ints6* was named *deleted in cancer 1* (*dice1*) and investigated as a putative tumor suppressor gene in humans [37], [38], [39], [40]. Using a forward genetic approach, we have revealed a novel role for Ints6 in limiting the extent of dorsal organizer tissues during vertebrate embryogenesis.

## Results

### A vertebrate recessive maternal-effect dorsalizing mutation

**Figure 1:**
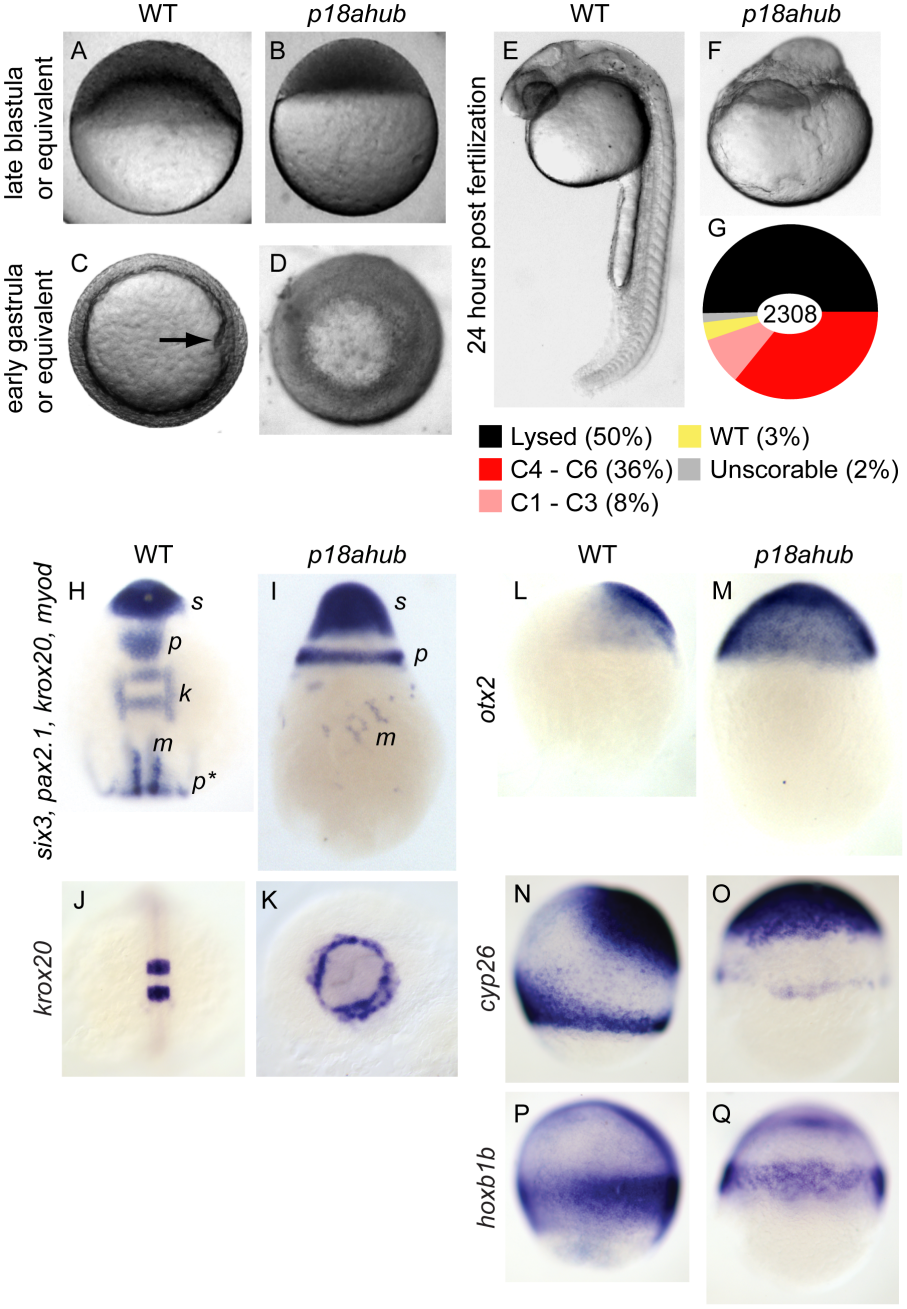
*p18ahub* mutants exhibit delayed progression of epiboly and severe dorsalization. Late blastula stage WT embryo (A), and age-matched *p18ahub* embryo (B). WT embryo at an early gastrula stage (C) with the shield (organizer) on the dorsal side (arrow). A *p18ahub* embryo at an early gastrula stage (D) displaying apparent radial hyperconvergence with no definitive dorsal side. (A,B) lateral views; (C,D) animal pole views. WT (E) and *p18ahub* (F) embryos at 24 hours post fertilization (hpf) (lateral views). (G) Pie chart depicting proportion of embryos with indicated phenotypes at 24 hpf sampled over a total of 2308 embryos. (H and I), *in situ* hybridization on 3–5-somite stage WT (H, n = 15) and age-matched *p18ahub* (I, n = 13) embryos for *six3* (*s*) expression in presumptive forebrain, *pax2.1* expression in the midbrain-hindbrain boundary (*p*) and pronephros (p*), *krox20* (*k*) in hindbrain rhombomeres 3 and 5, and *myod* (*m*) in paraxial mesoderm; dorsal view in (H), lateral view in (I), anterior to top. (J and K), 10-somite stage WT (J, n = 17, dorsal view), and age-matched *p18ahub* embryos (K, n = 18, anterior view) processed for *krox20 in situ* hybridization. (L–Q) *in situ* hybridization on mid gastrula stage WT and equivalent stage *p18ahub* embryos shown for: *otx2*, WT (L, n = 18) and *p18ahub* (M, n = 10); *cyp26a*, WT (N, n = 16) and *p18ahub* (O, n = 15); and *hoxb1b*, WT (P, n = 22) and *p18ahub* (Q, n = 21). Lateral views, anterior at top, dorsal to right.

We isolated *p18ahub*, a recessive maternal-effect dorsalizing mutation, in an ENU-induced mutagenesis screen designed to identify novel maternal factors required for early embryonic development and patterning in zebrafish (similar to that described in [41], [42]). Mutant females yielded embryos with similar phenotypes whether they were crossed to mutant or wild-type (WT) males, indicating a strictly maternal-effect defect with no zygotic contribution. The first defect evident in embryos from *p18ahub* mutant mothers (henceforth referred to as *p18ahub* mutant embryos) was a delay in the initiation of epiboly, the morphogenetic process by which the blastoderm cells move over and encompass the yolk cell [43]. As WT embryos reached the late blastula/50% epiboly stage (Figure 1A), mutant embryos typically had not initiated epiboly movements (Figure 1B). In early gastrulation stages, WT embryos displayed a single dorsal thickening corresponding to the embryonic axis (Figure 1C), whereas mutant embryos often developed a radial thickening possibly due to hyper convergence of cells around the entire embryonic margin (Figure 1D). Approximately 50% of mutant embryos (Figure 1G) lysed prior to 24 hpf.

Thirty-five percent of mutant embryos surviving to 24 hours post fertilization (hpf) exhibited radial symmetry around the animal-vegetal axis and lacked any recognizable structures (Figure 1E,F). We categorized such embryos as class 6 dorsalized embryos. Class 5 embryos lacked recognizable structures but were not radially symmetric around the animal-vegetal axis and typically did not survive to 24 hpf. Class 4 through class 1 embryos (Figure 1G) exhibited progressively less severe dorsalization, as described [5]. To simplify the presentation of the data, we have combined phenotypic classes, C1–C3, and, C4–C6, in all figures, except that C5 embryos that did not survive to 24 hpf are included in a lysed category.

To better examine the epiboly and lysis defects of *p18ahub* embryos, we conducted time-lapse imaging (supplemental Movie S1 and Movie S2) of embryos from mid-blastula to mid-somitogenesis stages. *p18ahub* embryos were developmentally delayed and in some mutant embryos displayed prolonged epiboly (Movie S2). Some *p18ahub* embryos failed to undergo epiboly whatsoever and lysed by the equivalent of early gastrula stage in WT, with cells dispersing rapidly and the blastoderm disintegrating (Movie S1). In other *p18ahub* embryos, epiboly progressed to just prior to yolk plug closure (100% epiboly) when the hypoblast rapidly retracted and either the embryo lysed or the tissue dived down into the animal pole of the yolk cell (Movie S2), accounting for the morphology of embryos like the one shown in Figure 1F. Based on these studies it is likely that the lysed *p18ahub* embryos for a given clutch displayed either the early lysis phenotypes or were class 5 or 6 dorsalized.

To investigate if *p18ahub* embryos exhibit altered DV patterning, we examined the expression of genes of the dorsally-derived neurectoderm, dorsolaterally-derived somitic and paraxial mesoderm, and ventrally-derived pronephros, by *in situ* hybridization on 3- to 5-somite stage embryos. The expression of both *six3*, which marks forebrain neurectoderm [44], and *pax2.1*, a marker of the midbrain-hindbrain boundary [45], was circumferentially expanded in *p18ahub* embryos (Figure 1H,I). *krox20*, which is expressed in rhombomeres 3 and 5 in the hindbrain [46], was often undetectable in severely dorsalized *p18ahub* embryos due to their severe delay (8/23 embryos displayed *krox20* expression for the clutch represented in Figure 1I). In *p18ahub* embryos able to develop longer, *krox20* expression was also expanded circumferentially (Figure 1J,K).

The expression of *myod*, a marker of paraxial and somitic mesoderm [47], was scattered in clusters of cells distributed circumferentially (Figure 1I, verified by examination of *myod* probe alone, not shown). Pronephric *pax2.1* expression was often undetectable in mutant embryos (compare Figure 1I p* with H; verified by examination of *pax2.1* probe alone, not shown), indicative of a severe reduction of ventroposterior mesoderm.

To investigate if patterning is also affected during gastrulation in *p18ahub* embryos, we examined the expression of the fore- and mid-brain marker *otx2* [48] at mid gastrulation. We found that *otx2* expression was expanded around the DV axis rather than restricted to a dorsoanterior region as in WT (Figure 1L and M). Importantly, however, *otx2* was not expanded posteriorly as in *wnt8a* mutants [18], suggesting that the Wnt8a pathway is intact in *p18ahub* embryos. We also examined the expression of *cyp26a* and *hoxb1b*, markers of anterior neurectoderm and caudal hindbrain, respectively [49], [50]. Consistent with dorsalization, *p18ahub* embryos displayed expanded *cyp26a* expression around the DV axis (Figure 1N,O). Note also that the *p18ahub* embryos were delayed developmentally; the margin of the mutant embryo (the equivalent time of bud stage for WT) has not advanced as far as in WT. Compared to WT (Figure 1N), the *p18ahub* embryo displays reduced marginal *cyp26a* expression, which is consistent with the WT *cyp26a* expression pattern at earlier gastrula stages [50]. Expression of *hoxb1b* in the posterior neurectoderm is expanded in *p18ahub* embryos around the margin, although with reduced intensity compared to WT (Figure 1P,Q), likely also reflecting developmental delay [49]. It was necessary to age-match embryos for these experiments because we could not obtain sufficient numbers of *p18ahub* embryos at mid-gastrula stage due to their lysis. These data indicate that *p18ahub* embryos display an expansion of dorsal neurectodermal cell fates during gastrulation, but unlike zygotic *wnt8a* mutants, no significant expansion of anterior at the expense of posterior neurectoderm is observed in *p18ahub* embryos.

### Although severely repressed, BMPs can signal in *p18ahub* embryos

**Figure 2:**
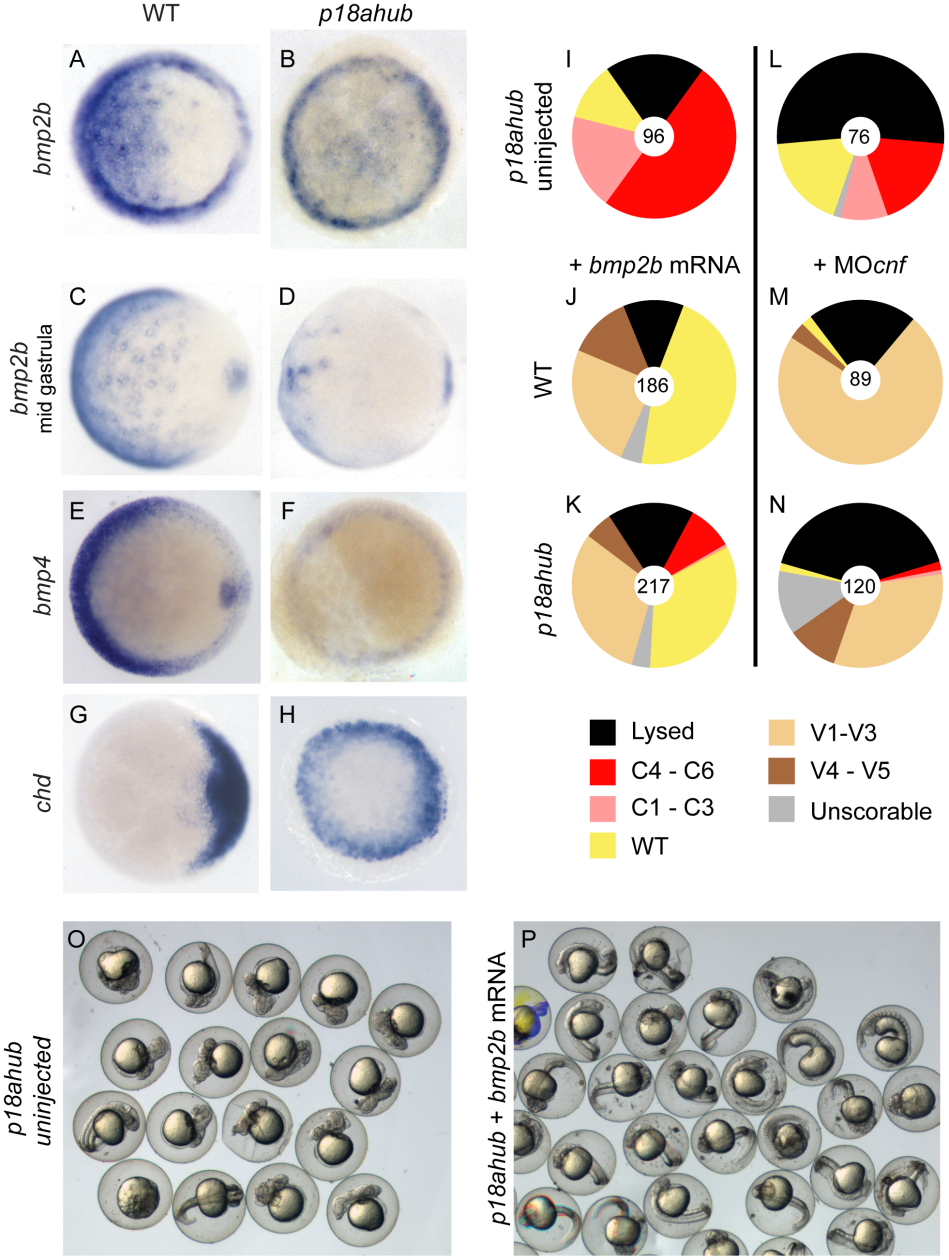
Reduced *bmp* expression but intact BMP signal transduction in *p18ahub* mutants. (A–H) *In situ* hybridization on early gastrula stage embryos or equivalent, except (C) and (D) are mid gastrula stage or equivalent. Embryos were stage-matched. Animal pole views, dorsal to the right for all. Expression of *bmp* ligand gene in WT embryos (A, n = 32, C, n = 43, and E, n = 21) and *p18ahub* embryos (B, n = 30, D, n = 18, and F, n = 16). Expression of the BMP antagonist *chordin* (*chd*) was confined to the dorsal side of WT embryos (G, n = 21) but was expanded around the entire margin of *p18ahub* embryos (H, n = 20) by early gastrulation. (I–N) Pie charts indicate fractions of embryos at 24 hpf that displayed the indicated phenotypes (categories described in the text). Number of embryos is shown at the center of each chart. *p18ahub* embryos (I, O, uninjected) that were injected with 20 pg *bmp2b* mRNA were rescued to WT or ventralized (K, P, +*bmp2b* mRNA) similarly to WT embryos (J, +*bmp2b* mRNA). *p18ahub* mutant embryos (L, uninjected) depleted of Chordin, Noggin1, and Follistatin-like 2b proteins by MO injection were similarly ventralized (N, +MO*cnf*) to WT embryos (M, +MO*cnf*).

To determine if the dorsalization of *p18ahub* embryos results from impaired BMP signaling, we examined BMP ligand gene expression, as well as expression of the BMP antagonist *chordin* (*chd*) in mutant embryos. *bmp2b* expression appeared normal in late blastula stage mutant embryos (data not shown). However, *bmp2b* and *bmp4* expression were significantly reduced in mutant embryos by the early gastrula stage (Figure 2A,B,E,F) and severely reduced by mid gastrulation (Figure 2C, D, and data not shown), consistent with dorsalization. Since BMP gene expression is controlled by an autoregulatory feedback loop [3], , the loss of *bmp2b* and *bmp4* expression in mutant embryos indicates severely reduced BMP signaling. *chd* expression, which is restricted to the dorsal side of the early zebrafish gastrula [53], is circumferentially expanded in mutant embryos at an early gastrula stage (Figure 2G,H), consistent with reduced BMP signaling and excessive dorsal fate specification in *p18ahub* embryos.

To determine if *p18ahub* embryos are defective mechanistically in BMP signal transduction, we injected mutant embryos with *bmp2b* mRNA, which moderately to severely ventralizes WT embryos [6] (Figure 2J). We found that forced expression of *bmp2b* restored ventral fates to WT in two-thirds of mutant embryos or moderately ventralized them (Figure 2I,K,O,P). Thus, the BMP signal transduction machinery in mutant embryos can function when BMP ligand is provided exogenously.

To further test whether the endogenous BMP signaling machinery is functional in *p18ahub* embryos, including the endogenous BMP ligands, mutant embryos were injected with translation-blocking morpholinos (MOs) targeting the secreted BMP antagonists Chordin, Noggin1, and Follistatin-like 2b [54]. Depletion of these BMP antagonists in mutant embryos caused mild to moderate ventralization, similar to that of WT embryos (Figure 2M,N). Thus, endogenous BMP ligands can signal in *p18ahub* embryos at a WT or greater level if BMP ligand function is permitted.

### Organizer genes are derepressed in *p18ahub* embryos

**Figure 3:**
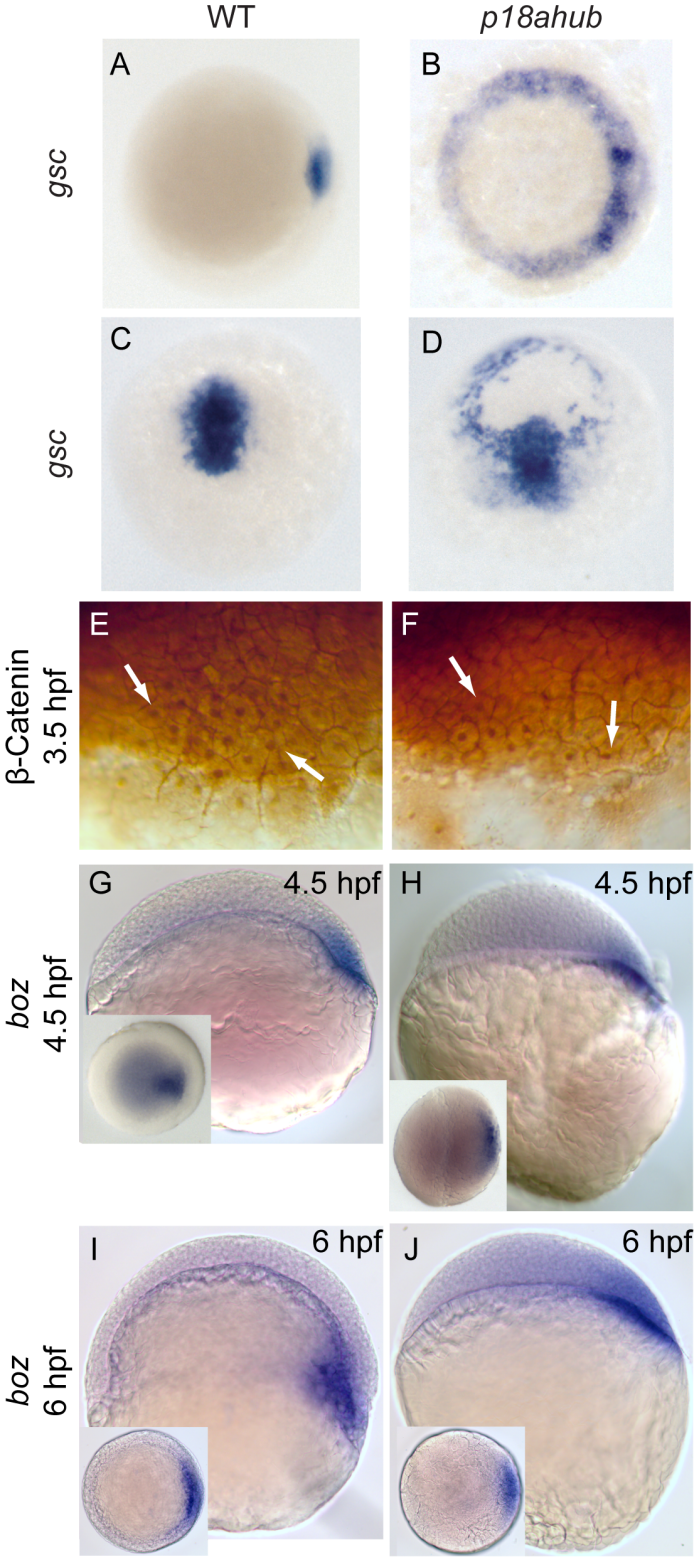
Dorsal organizer gene expression is expanded in *p18ahub* mutants. *In situ* hybridization using *goosecoid* (*gsc*) probe (A–D). Animal pole views, dorsal to right for (A) and (B); dorsal view, animal pole up in (C) and (D). WT embryos at early gastrula (A, n = 27) and mid gastrula stages (C, n = 41). *p18ahub* embryos (B, n = 33 and D, n = 10) displayed radially expanded *gsc* expression at the same stages. The embryo in (D) is tilted toward the viewer to show radial *gsc* expression. WT (E, n = 18) and *p18ahub* (F, n = 18) blastula stage embryos showed no obvious differences in the number or distribution of β-catenin immunopositive nuclei (arrows). (G–J) *In situ* hybridization for *bozozok (boz)*, lateral views, dorsal to right; insets show animal pole views. WT and *p18ahub* embryos showed normal *boz* expression at late blastula (G, n = 19 and H, n = 18) and early gastrula stages (I, n = 14 and J, n = 16, respectively). As described in the text, epiboly progression was delayed in *p18ahub* embryos. At 6 hpf WT embryos form a morphologically apparent organizer, indicated by a thickening of the blastoderm on the dorsal side of the embryo and *boz* expression within the hypoblast. In contrast, at 6 hpf *p18ahub* embryos still appeared to be in a late blastula stage, although their *boz* expression was similar to WT.

Dorsalization can also be caused by a ventral expansion of dorsal organizer gene expression. To investigate the organizer in *p18ahub* mutant embryos, we examined expression of the organizer gene *goosecoid (gsc)* [55]. We found that, although *gsc* expression was induced normally at the mid blastula stage (data not shown), it was expanded in *p18ahub* embryos by early gastrulation (Figure 3A,B) and remained ectopically expressed through mid gastrula stages (Figure 3C,D) compared to WT embryos. Therefore, the dorsalization of *p18ahub* embryos involves a prominent expansion of *gsc* expression by early gastrulation, contrasting dorsalization resulting solely from defective BMP signaling [5].

### Wnt-mediated organizer induction is normal in *p18ahub* embryos

Since *gsc* expression was induced normally in *p18ahub* mutant embryos, it suggested that the organizer is induced normally by the maternal Wnt signaling pathway [1], [2]. To directly test this, we examined β-catenin nuclear localization as a readout of the maternal Wnt signaling pathway. We immunostained mid-blastula stage embryos (3.5 hpf) to visualize β-catenin in nuclei on the presumptive dorsal side of the embryo [1]. No differences were evident between WT and mutant embryos in β-catenin intensity or its localization in nuclei at the dorsal margin (Figure 3E, F, arrows). No nuclear localized β-catenin was observed ventrally in mutant embryos. Consistent with normal induction of the organizer, *boz*, a direct transcriptional target of the maternal Wnt pathway [56], was expressed normally in mutant embryos through 6 hpf, the equivalent of early gastrula stage (Figure 3G–J), unlike *gsc* and *chd*, which were expanded (Figure 2H and 3B). Thus, the organizer is induced normally in mutant embryos but the expression of some organizer genes becomes derepressed around the margin between late blastula and early gastrula stages. Furthermore, the dorsalization of *p18ahub* embryos does not rely upon ventrolateral expansion of *boz*.

### The Wnt8a pathway is functionally intact but repressed in *p18ahub* embryos

**Figure 4:**
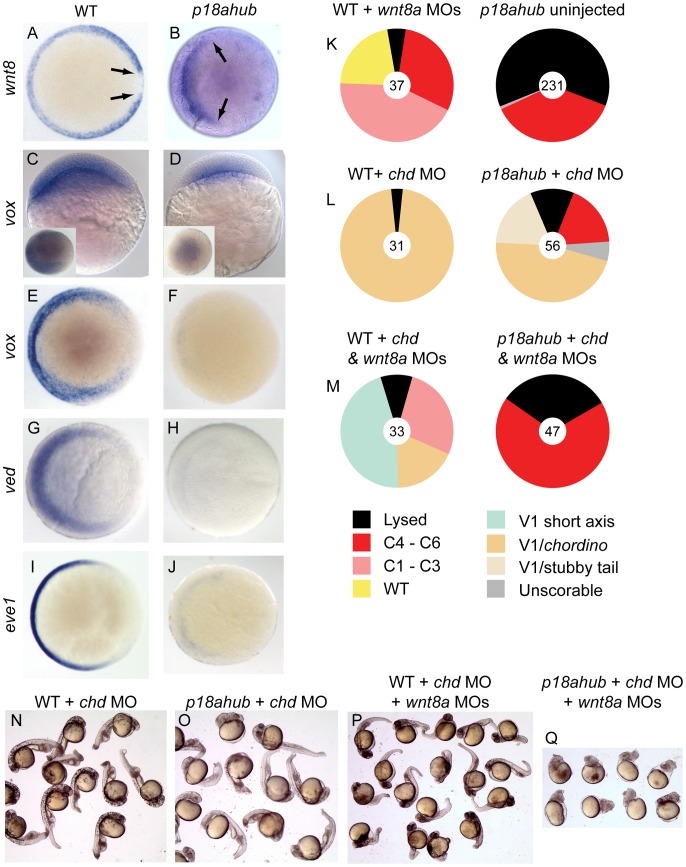
The Wnt8a pathway is repressed but mechanistically intact in *p18ahub* embryos. (A–J) *In situ* hybridization for Wnt8a pathway components or downstream genes. Animal pole views, dorsal at right, except (C, D) are lateral views, dorsal at right, insets show animal pole views. At an early gastrula stage in WT (A, n = 14) *wnt8a* was expressed around the margin but was absent from the axial mesoderm (arrows). In *p18ahub* embryos at a late blastula stage (B, n = 20) *wnt8a* expression was absent from the presumptive dorsal half of the embryo. The expression of *vox* was reduced in late blastula *p18ahub* embryos (D, 11/13 embryos) compared to WT embryos (C, n = 18). By early gastrulation, WT embryos (E, n = 20) showed prominent ventrolateral *vox* expression, while *vox* was nearly absent from equivalent stage *p18ahub* embryos (F, n = 12). *ved* was expressed similarly to *vox* in WT (G, n = 10) and was also nearly undetectable in *p18ahub* embryos (H, n = 10) at early gastrulation. *eve1* in WT (I, n = 17) marks developing ventroposterior mesoderm and was nearly absent from *p18ahub* embryos by early gastrulation (J, n = 12). Splice blocking *wnt8a* MOs variably dorsalized WT embryos (K). (L) Injection of MO against *chd* ventralized both WT and *p18ahub* embryos (compare to *p18ahub* uninjected, K). (M) Simultaneous reduction of Chd and Wnt8a causes dorsalization in *p18ahub* embryos, indicating that endogenous Wnt8a signal transduction is intact and functional in *p18ahub* embryos that are depleted of Chd. Representative embryos at 1 dpf are shown in N–Q.

Zygotic Wnt8a signaling in ventrolateral regions represses the expression of the organizer genes, *gsc* and *chd*, thus restricting the size of the organizer [7], [8], [9], [10], [11], [18], [19], [26]. Loss of Wnt8a or its mediators Vox, Vent, and Ved causes the expression of some organizer genes to expand and dorsalizes the embryo [7], [8], [9], [10], [11], [19]. Accordingly, we investigated the status of the Wnt8a pathway in mutant embryos. In WT late blastula and early gastrula stage embryos, *wnt8a* is expressed at the margin in a large domain extending from ventral to dorsolateral regions (Figure 4A) [18], [57]. However, in *p18ahub* late blastula stage embryos, *wnt8a* expression was restricted ventrally to a smaller domain (Figure 4B). Furthermore, *wnt8a* expression was undetectable in mutant embryos age-matched to their WT counterparts at early gastrula stage (not shown), perhaps reflecting a loss in the competence of marginal cells to express *wnt8a*. Since *wnt8a* is required for anteroposterior (AP) patterning of neural tissue [18] and a defect is not evident in AP patterning in *p18ahub* mutants, it suggests that the early, transient expression of *wnt8a* may be sufficient for AP patterning in *p18ahub* mutant embryos.

We also examined the expression of *vox* and *ved*, two genes encoding transcriptional repressors acting downstream of Wnt8a to restrict organizer gene expression to the dorsal midline [10], [11], [19]. We found that *vox* expression was reduced in mutant embryos compared to age-matched WT embryos at a late blastula stage (Figure 4C,D). By early gastrulation, *vox* expression was greatly reduced or absent in mutant embryos (Figure 4E,F). We also conducted a time course experiment where embryos were collected from WT and *p18ahub* females over half hour intervals beginning at a mid-blastula stage (3 hpf) and subsequently examined for *wnt8a* and *vox* expression. In this experiment induction of neither gene was observed in *p18ahub* embryos by the equivalent of the early to mid gastrula stage (7 hpf) in WT (not shown). Maternal *ved* expression was evident in *p18ahub* embryos (data not shown); however, by an early gastrula stage *ved* expression was prominently reduced (Figure 4G,H). Early gastrula stage embryos also displayed reduced or nearly absent expression of *eve1*, a marker of ventroposterior mesoderm that requires BMP and Wnt8a signaling for its expression (Figure 4I,J) [58], [59], [60]. Thus, key mediators of the Wnt8a pathway are repressed in *p18ahub* embryos beginning as early as the late blastula stage, which can account for the loss of ventroposterior mesoderm.

We could not restore Wnt8a signaling in mutant embryos via injection of *wnt8a* mRNA, because early overexpression of *wnt8a* mimics the maternal Wnt signal for organizer induction and severely dorsalizes embryos [57]. To determine if the zygotic Wnt8a signaling pathway is functional in *p18ahub* embryos, we tested for Wnt8a function in *p18ahub* embryos rescued by depletion of BMP antagonists. If Wnt8a signaling is required, then it would indicate that the pathway is functional but likely fails to function in *p18ahub* embryos due to lack of full induction or maintenance of expression. To moderately increase BMP signaling in *p18ahub* embryos, we depleted one BMP antagonist, Chd, via MO injection. Loss of Chd alone rescued the majority of *p18ahub* embryos to a mild V1 ventralized phenotype, similar to loss of Chd in WT embryos (Figure 4K,L,N,O). Loss of *wnt8a* dorsalized WT embryos [18] (Figure 4K) and also dorsalized *chd* deficient embryos or caused posterior truncations (Figure 4M,P). Importantly, *p18ahub* embryos that were enabled to specify ventral tissues by Chd depletion became dorsalized again when Wnt8a was also depleted (Figure 4K–M,O,Q). These results indicate that the Wnt8a pathway is mechanistically intact in *p18ahub* embryos and that their ventralization or rescue to a WT phenotype by enhancement of BMP signaling depends on endogenous Wnt8a signaling.

### Axial mesoderm is excessively specified at the expense of ventrolateral mesoderm in *p18ahub* embryos

**Figure 5:**
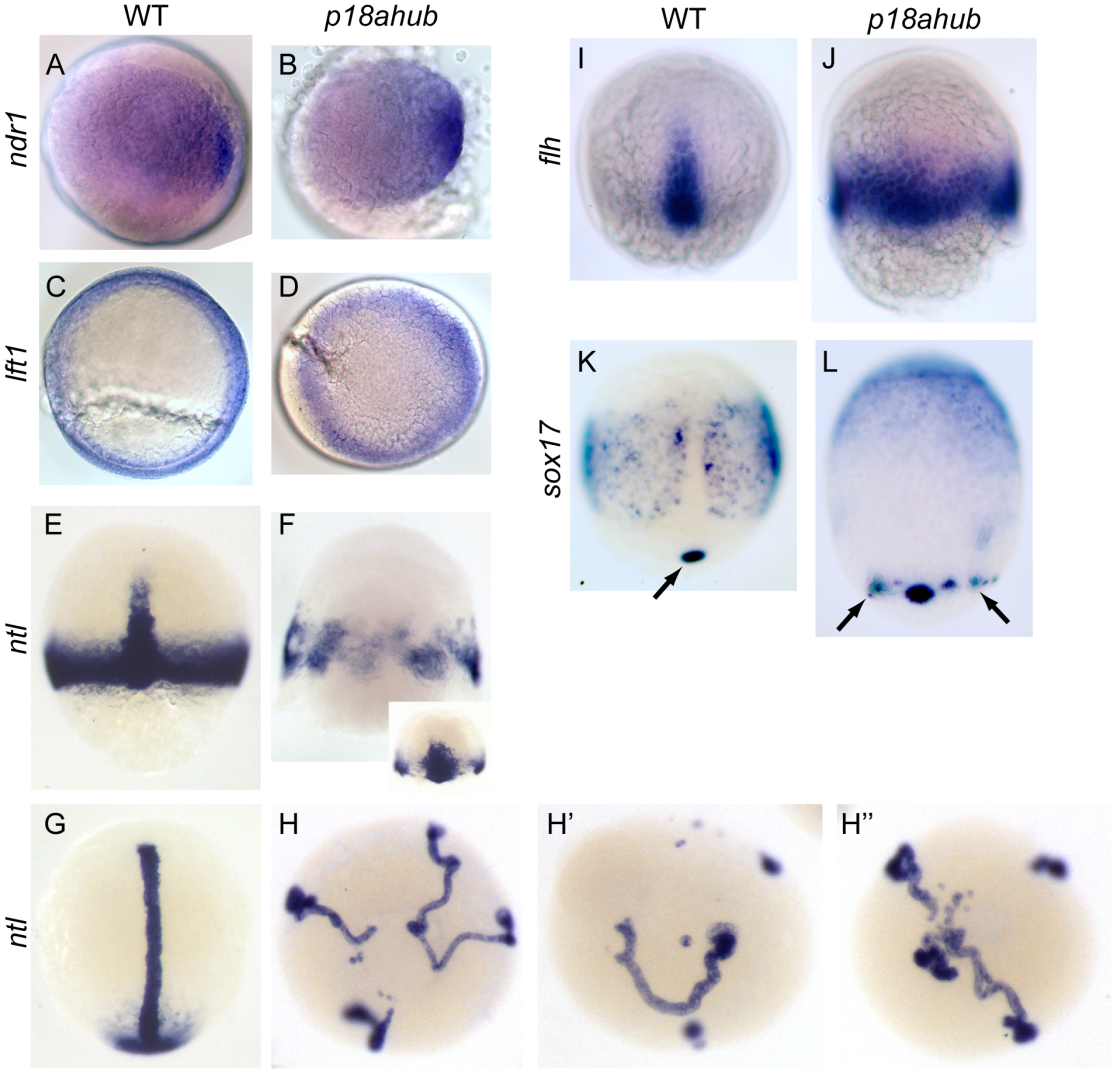
Excessive axial mesoderm at the expense of ventrolateral mesoderm in *p18ahub* embryos. WT (A, n = 19) and stage-matched *p18ahub* (B, n = 20) embryos at a mid blastula stage, exhibited similar *nodal related 1 (ndr1)* expression. WT (C, n = 14) and age-matched *p18ahub* (D, n = 13) embryos at 6 hpf after synchronized matings displayed similar *lefty1* (*lft1*) expression, although blastoderm involution was not evident in the mutants. (A–D) are animal pole views, dorsal to right. (E–L) are mid gastrula stage, except (G and H) are 3–5 somite stage or equivalent. Dorsal views, animal to top, except (H–H″) are animal pole views. WT embryos (E, n = 6) displayed marginal and axial *ntl* expression. In contrast, some *p18ahub* embryos displayed only axial *ntl* expression distributed circumferentially (F, n = 16), while others displayed broadened axial and reduced ventrolateral *ntl* expression (F inset, n = 8). In WT embryos (G, n = 22) *ntl* was expressed in the notochord and tail bud mesenchyme. In equivalent stage *p18ahub* embryos, (H–H″, n = 21), *ntl* expression was observed in multiple notochords terminating in smaller tail bud-like domains. Consistent with ectopic axial *ntl* expression, the expression of *floating head* (*flh*), a marker of notochord precursors, confined dorsally in WT embryos (I, n = 20), was expressed around the entire embryonic margin in *p18ahub* embryos (J, n = 16). In WT (K, n = 18) *sox17* is expressed in endodermal precursor cells and in a single cluster of dorsal forerunner cells (arrow). In *p18ahub* embryos (L, n = 16) *sox17* expression indicates the presence of ectopic clusters of dorsal forerunner cells (arrows).

Along with the maternal Wnt pathway, the dorsal organizer also depends on Nodal signaling for its induction (reviewed in [61]). Thus, we investigated the status of the Nodal pathway in *p18ahub* embryos. We examined expression of the Nodal ligand *nodal-related 1* (*ndr1, squint*) in mutant and WT embryos [62]. *ndr1* induction is initiated on the dorsal side of the embryo (Figure 5A) and requires Wnt signaling similarly to *boz* [2]. At a mid blastula stage, we observed no significant differences in the expression of *ndr1* between WT and *p18ahub* embryos (Figure 5B) and *ndr1* expression was never observed more animally outside of its normal marginal domain through an early gastrula stage equivalent (not shown).

To further test if Nodal signaling is induced normally in *p18ahub* embryos, we examined expression of the Nodal feedback antagonist *lefty1 (lft1, antivin1)*. *lft1* is initially expressed dorsally but is subsequently expressed around the margin of the late blastula [63], [64], [65]. At an early gastrula stage (6 hpf), we observed no significant differences in the expression level of *lft1* in mutant versus WT embryos (Figure 5C and D). From these data we conclude that Nodal signaling in *p18ahub* embryos is induced normally and likely operates normally at an early gastrula stage.

By mid gastrula stages the pan-mesodermal gene *no tail* (*ntl*) is expressed in two distinct domains, one corresponding to axial mesoderm, the developing notochord, and another corresponding to ventrolateral non-axial mesoderm [66]. At the equivalent of mid gastrula and early somite stages, *ntl* expression in presumptive non-axial mesoderm was reduced or absent, while axial mesoderm appeared to be expanded leading to multiple independent axes in some mutant embryos (9/9, 6/10, and 4/10 in three independent clutches) (Figure 5E–H″). Some mutant embryos displayed a significantly broadened single axial *ntl* domain with a prominent reduction in marginal *ntl* expression (Figure 5F inset). By the equivalent of early somite stages of development, mutant embryos clearly possessed multiple independent presumptive notochords marked by *ntl* expression (Figure 5H).

*floating head* (*flh*), a homeobox gene required for notochord specification, is induced at a late blastula stage independently of *ntl* but is a direct transcriptional target of *ntl* by mid gastrula stages [67], [68], [69]. Consistent with excessive axial *ntl* expression, *flh* was also circumferentially expanded in *p18ahub* embryos by mid gastrulation (Figure 5I,J). Therefore, genes marking axial mesoderm (*gsc* and *flh*) are ectopically expressed in *p18ahub* embryos by early gastrula stage, despite normal Wnt-mediated organizer induction and normal induction of the Nodal pathway.

### *p18ahub* embryos display ectopic dorsal forerunner cells

Nodal signaling is required for the expression of the HMG- type transcription factor *sox32* (*casanova* (*cas*)) in endodermal precursors by late blastula stages [35], [70]. *cas* is required along with *pou5f3* (formerly *spiel-ohne-grenzen, pou5f1*) to induce *sox17* and maintain endodermal precursor cells [21]. Both *cas* and *pou5f3* are also required for the maintenance of dorsal forerunner cells [21], [33], a distinct population of non-involuting cells that also express *sox17* at the leading edge of the dorsal margin of the blastoderm as it migrates over the yolk [34], [71]. We observed endodermal *sox17* expression in *p18ahub* embryos at mid gastrulation (Figure 5L). However, endodermal precursor cells were located more animally than in WT (Figure 5K), perhaps due to altered cell movements resulting from loss of BMP signaling and dorsalization [72], [73]. Ectopic marginal expression of *sox17* was observed in *p18ahub* mid gastrula stage embryos indicating that ectopic dorsal forerunner cells form in mutant embryos (Figure 5K,L, arrows).

In *p18ahub* embryos axial mesoderm and organizer gene expression are expanded ventrolaterally, but remain confined within a normal mesodermal domain. Importantly, we did not observe the animal-ward expansion of Nodal-dependent genes such as *ntl* or *lft1* or excessive specification of mesendodermal precursor cells, which has been reported upon excessive Nodal signaling due to loss of Lefty [74], [75], [76] or ectopic expression experiments [77], [78]. Thus, Nodal signaling is likely not excessive in *p18ahub* embryos.

### Suppression of Nodal signaling rescues patterning of *p18ahub* embryos

**Figure 6:**
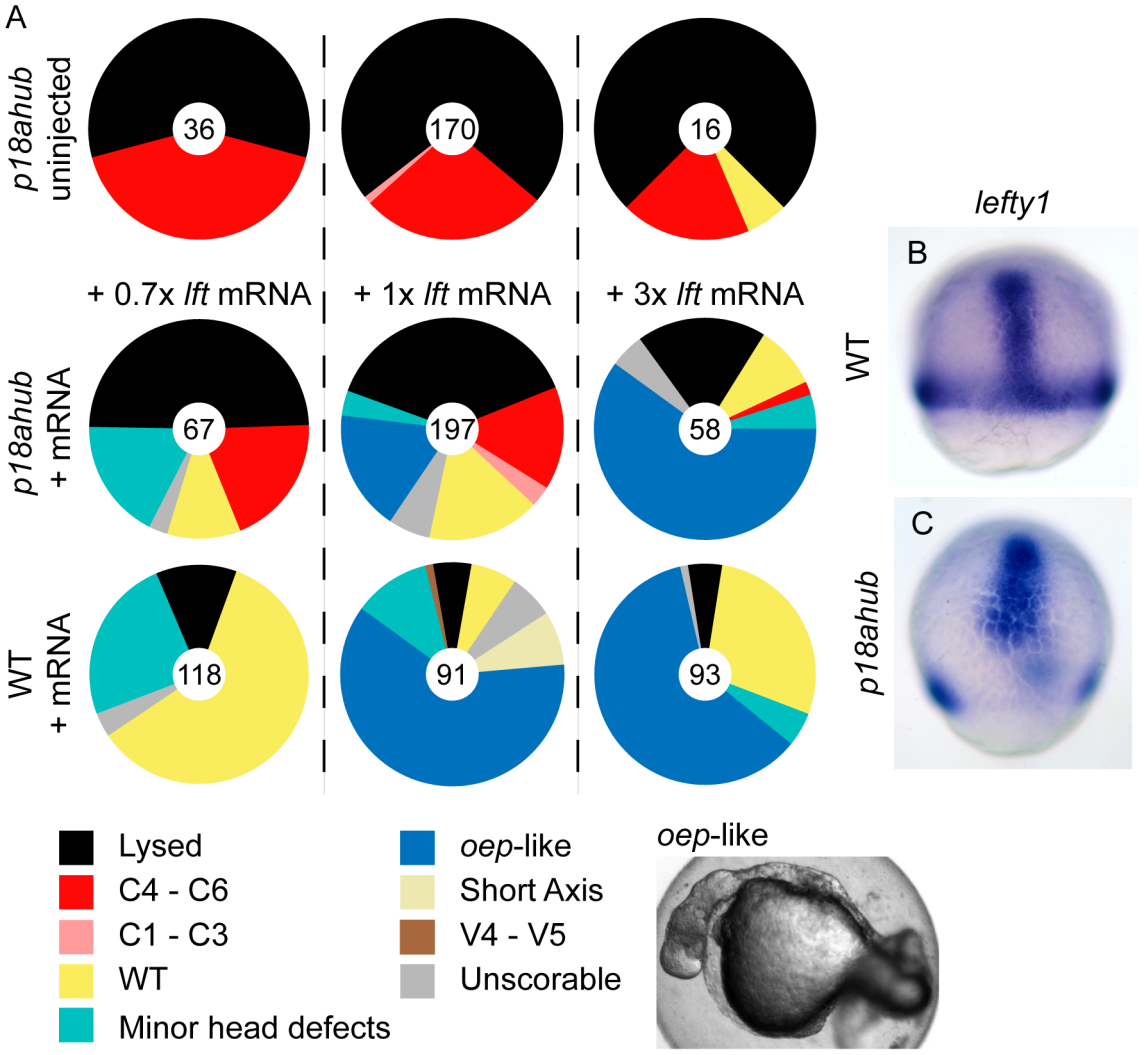
Lefty misexpression suppresses patterning defects of *p18ahub* embryos. (A) Pie charts indicate fractions of embryos at 24 hpf with the indicated phenotypes. Number of embryos is at the center of each chart. Labels across indicate the amount of *lefty1 (lft1)* mRNA injected. Top row shows uninjected *p18ahub* control clutch for each experiment, middle row indicates *p18ahub* embryos injected with mRNA, and bottom row is WT embryos injected with mRNA. +1× *lft* mRNA corresponds to 1 pg mRNA. (B and C) *In situ* hybridization for *lft1* in a mid gastrula stage WT (B, n = 12) and *p18ahub* (C, n = 13) embryos; lateral views, dorsal facing.

The secreted feedback inhibitor Lefty/Antivin (Lft1) regulates Nodal signaling [63], [64], [65], [79]. Misexpression of Lft1 in WT embryos severely limits mesendoderm induction with embryos closely resembling *ndr1*;*ndr2* double mutants [62]. We found that injection of as little as 0.7 picograms (pg) of *lft1* mRNA (Figure 6A, +0.7× middle row), a dose that only weakly perturbs WT embryos (Figure 6A, *Minor head defects*), could restore WT or nearly WT patterning in 33% of mutant embryos. Injection of 1 pg of *lft1* mRNA (Figure 6A, +1× middle row) restored WT patterning in a larger fraction of mutant embryos and suppressed mesendoderm formation (*oep*-*like*) in 20% of mutant embryos. The same dose of *lft1* mRNA injected into WT embryos blocked mesendoderm development in more than 50% of the embryos (Figure 6A, +1× bottom). Injection of 3 pg of *lft1* mRNA into mutant and WT embryos inhibited mesendoderm induction in a similar fraction of embryos (Figure 6A, +3×). Thus, suppression of Nodal signaling can restore the balance between axial and non-axial fate specification in mutant embryos, similarly to restoring BMP signaling.

We examined *lft1* expression to determine if its reduction contributed to excess dorsal mesodermal gene expression at mid gastrulation in *p18ahub* embryos. In WT embryos *lft1* was expressed around the embryonic margin as well as in the developing axis and dorsal forerunner cells (not shown) at mid gastrula stages (Figure 6B). In *p18ahub* embryos *lft1* was expressed within the presumptive prechordal plate as well as in clusters of cells scattered around the margin at a mid gastrula stage (Figure 6C). These latter cells may represent remaining marginal cells having non-axial fates. Hence, an absence of *lft1* expression cannot account for the severe dorsalization of *p18ahub* embryos.

### *p18ahub* affects a highly conserved component of the Integrator Complex

**Figure 7:**
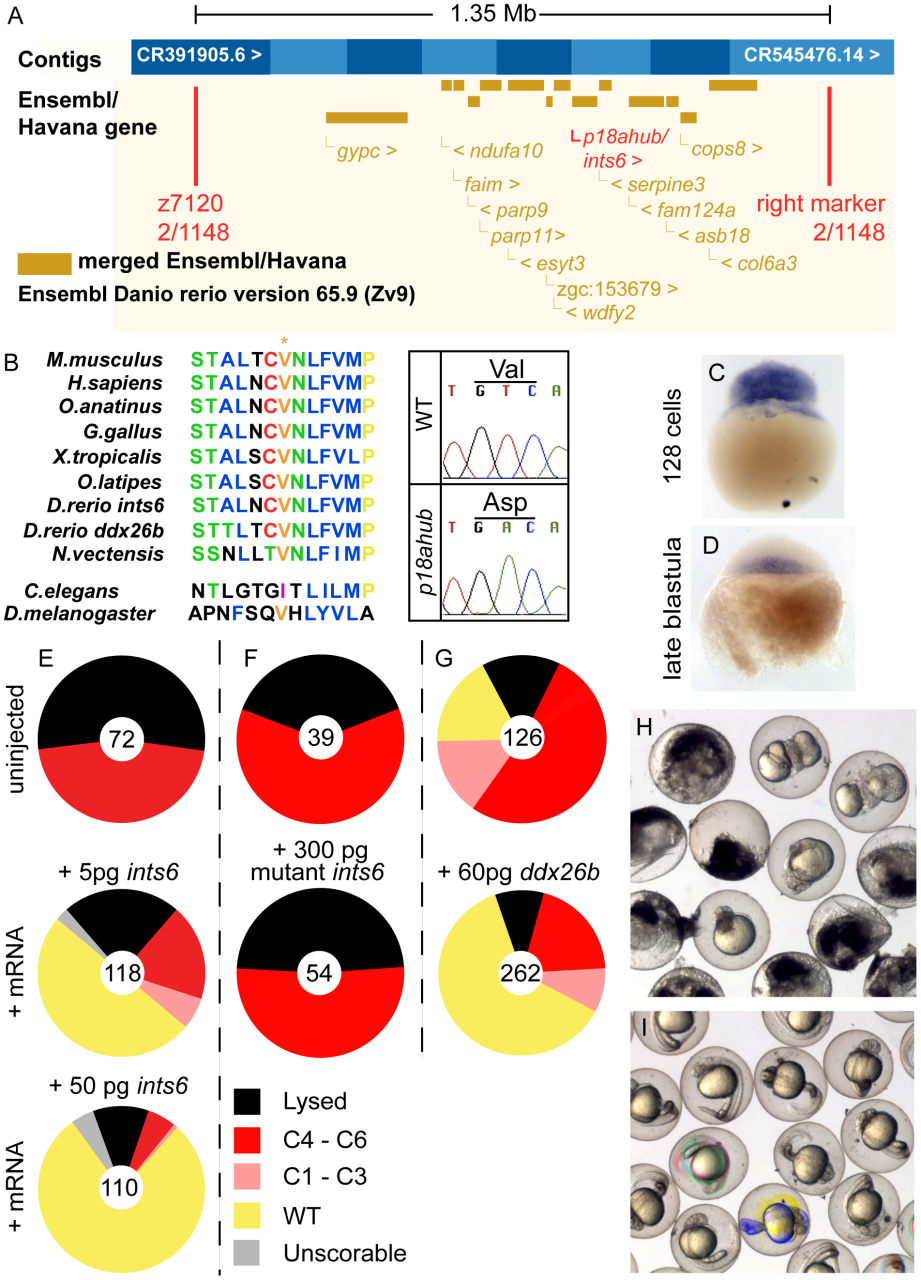
*p18ahub* affects the Integrator Complex Subunit 6 (Ints6). (A) *p18ahub* was narrowed to an approximately 1.35 Mb interval between SSLP marker z7120 and an SSLP marker that we generated in BAC CR545476.14 (Zv9) (right marker) by examining meiotic recombination between markers flanking the mutation. The recombinants found in the number of meioses examined at each marker is shown and the positions of markers are approximate. (B) *p18ahub* is a T to A transition in an exon of *ints6* that converts valine 375 to an aspartate. An alignment of several Ints6 proteins was generated using ClustalW and reveals that V375 is nearly invariant among widely divergent species. (C and D) *In situ* hybridization for *ints6* transcripts on WT embryos at (C) 128-cell (n = 8) and (D) late blastula stage (n = 19); lateral views, animal to top. (E–G) Rescue experiments: pie charts show fractions of embryos evaluated at 1 dpf with indicated phenotypes. Numbers of embryos are shown in the center of each chart. (H) Uninjected *p18ahub* mutant embryos and (I) mutant embryos rescued by injection of 50 pg *ints6* mRNA, shown at 1 dpf.

To identify the molecular nature of *p18ahub*, we mapped the mutation to a chromosomal position by examining linkage to simple sequence length polymorphic (SSLP) markers. We first found linkage of *p18ahub* to z1660 on chromosome 9. Further fine mapping examining over 1100 meioses placed *p18ahub* within a 1.35 Mb interval between the SSLP marker z7120 and an SSLP marker that we generated for BAC CR545476.14 (Zv9). (Figure 7A). The interval displays synteny with human chromosome 13. It contains just over one dozen genes, none of which were known to function in DV patterning. We proceeded to sequence the open reading frames of cDNAs of genes within the interval.

We found a T to A transition in an exon of the *integrator complex subunit 6 (ints6)* gene (GenBank Accession number KF700696, OMIM 604331), converting a nearly invariant valine to an aspartate at position 375 of the 854 amino acid predicted protein (Figure 7B). Human and zebrafish Ints6 orthologs are 66% identical indicating an overall high degree of evolutionary conservation. The only recognizable domain in Ints6 is an N-terminal von Willebrand factor type A motif (InterPro IPR002035), a broadly employed motif mediating interactions between diverse proteins. There is a second gene, *ddx26b*, on the X chromosome in humans and on chromosome 14 in zebrafish that is highly related to *ints6*. The zebrafish Ints6 and Ddx26b proteins are 61% identical also implying significant conservation of function between these homologs.

We examined the expression of *ints6* by *in situ* hybridization. *ints6* transcripts were present in eggs and embryos through the late blastula stage (Figure 7C,D, data not shown). *ints6* transcripts declined during late blastula stages and were undetectable by early gastrulation (Figure 7, data not shown). We observed no alteration in the expression of *ints6* in mutant embryos by *in situ* hybridization (not shown). These expression data are consistent with the recessive, maternal-effect inheritance of *p18ahub* and the maternal requirement for *ints6*.

### Ints6 is the gene affected by *p18ahub*

To determine if dorsalization of *p18ahub* embryos was caused by the mutation in *ints6*, we injected mutant embryos with mRNA encoding WT Ints6. We found that as little as 5 pg of WT *ints6* mRNA rescued 50% of mutant embryos completely (Figure 7E) and 50 pg rescued nearly 80% of mutant embryos to WT (Figure 7E,H,I). Thus, *ints6* is the defective gene in *p18ahub* mutant embryos. We also injected mutant embryos with up to 300 pg of different preparations of *p18ahub* mutant *ints6* mRNA and never detected rescue (Figure 7F). Thus, *p18ahub* is likely a strong loss-of-function or null allele. Overexpression of *inst6* in WT embryos produced no phenotype (data not shown). The related zebrafish *ddx26b* mRNA also rescued *p18ahub* embryos (Figure 7G), although it is not provided maternally to the embryo (data not shown). We did not detect *ddx26b* expression via *in situ* hybridization on embryos through 24 hpf, although we were able to amplify cDNA corresponding to *ddx26b* from ovary RNA (not shown).

## Discussion

### Ints6 is a negative regulator of vertebrate organizer gene expression

We have identified a recessive maternal-effect mutation in *integrator complex subunit 6* (*ints6*), a highly conserved member of an RNA processing machine for which no specific role in vertebrate development was known. Importantly, to our knowledge the *p18ahub* mutation represents the first mutation of the *ints6* gene in any organism to be reported. Interestingly, the loss of *ints6* causes a recessive, maternal-effect dorsalization whereby dorsal midline axial fates are expanded, generating multiple dorsal axes at the expense of ventrolateral fates, suggesting an expansion of the dorsal organizer itself or of organizer gene expression mediating dorsal fate specification. The dramatic radial dorsalization of affected embryos is not caused by an expansion of the maternal Wnt-mediated induction of the organizer or the induction of Nodal signaling. In *ints6* mutant embryos axial mesoderm is expanded along the DV axis at late blastula stages but remains confined near the margin where mesendoderm is normally induced, indicating a DV patterning defect rather than a general expansion of mesoderm.

The Integrator Complex was identified as a complex of 12 subunits that co-purifies with Deleted in split hand/split foot 1 (DSS1) in human HEK293 cells [36]. The Integrator Complex associates with the C-terminal domain (CTD) of the large subunit of RNA polymerase II (RNAP) and has been implicated in 3′ end processing of the U1 and U2 snRNAs of the splicesosome [36]. Phosphorylation of serine 7 in the heptapeptide repeats present in the CTD directs the Integrator Complex-bound RNAP to snRNA genes rather than to the promoters of protein coding genes [36]. Prior to those studies, *ints6* was referred to as *deleted in cancer 1* and investigated as a putative human tumor suppressor gene, given the loss of its expression in several tumor-derived cell lines and tumor specimens from patients [38], [39]. It was shown that the promoter of *ints6* is subject to hypermethylation in transformed cells and overexpression of *ints6* can suppress the anchorage-independent growth of prostate tumor-derived and non-small cell lung carcinoma-derived cell lines [37], [40].

Although Ints6 is a member of a complex required to produce functional spliceosomal snRNAs [36], [80], the dorsalized phenotype of the *ints6* mutant in the zebrafish is not suggestive of a broad maternal or zygotic function in snRNA 3′ end formation. We can rescue the dorsalized mutant phenotype by altering a number of pathways required for embryonic patterning. Such success in our rescue experiments is difficult to reconcile with a widespread splicing defect. Since we can rescue dorsalization by injecting WT *ints6* mRNA, Ints6 functions in the embryo rather than during oogenesis, when maternal mRNAs are transcribed and spliced. Tao et al. have reported that MO-mediated knockdown of Integrator Complex subunits 5, 9, and 11 in the zebrafish results in defective hematopoiesis, possibly due to alteration of zygotic snRNA and mRNA processing [81]. The lysis defect that we observe in some early gastrula stage *p18ahub* embryos may reflect a more general function for Ints6, possibly in snRNA 3′ end formation or another unidentified role. In this regard *p18ahub* may be a hypomorphic allele that perturbs Ints6 function as part of the Integrator Complex.

The specific role of Ints6 within the Integrator Complex is unknown. RNAi-mediated knockdown of Ints6 in *Drosophila* S2 cultured cells causes low levels of splicing defects of snRNAs and of a U7-GFP reporter. These effects are orders of magnitude less than the alteration of snRNA processing reported in *Drosophila* S2 cells for RNAi-mediated knockdown of Ints9 or Ints11, putative catalytic subunits of the Integrator Complex [80]. It is also possible that the *p18ahub* missense mutation in *ints6* affects some but not all roles of Ints6 equivalently.

Prior to our work *C.elegans deleted in cancer 1* (*dic-1*) was the only *ints6* homolog to be investigated in any developmental context [82]. However, *C.elegans* DIC-1 is highly divergent from zebrafish Ints6 displaying only 26% identity and may not represent a true ortholog. *C.elegans* DIC-1 is required to maintain the viability of oocytes through oogenesis and embryonic viability in general, since RNAi depletion in embryos leads to arrested development and widespread cell death. No defects in embryonic patterning were reported [82].

### Early gastrula stage expansion of axial mesoderm in *ints6* mutant embryos

Nuclear β-catenin was normally localized dorsally in mid-blastula stage *ints6* mutant embryos indicating that maternal Wnt-mediated organizer induction is normal. Furthermore, *boz* expression was also normal in mutant embryos through the equivalent of an early gastrula stage [7], [11]. The expression of *ndr1*, *lft1*, and *ntl* in both WT and *ints6* mutant embryos was initially confined to the dorsal side of the embryo and expanded normally ventrolaterally around the blastoderm margin by late blastula stages [62], [63], [64], [83]. The earliest alteration of organizer gene expression that we observed in *p18ahub* embryos was the derepression of marginal *gsc* expression at an early gastrula stage. By mid gastrulation, *ntl* and *flh* expression in the axial mesoderm was also extensively expanded in mutant embryos.

Several lines of evidence distinguish the induction of the Nodal pathway in the axial mesoderm from its induction throughout the margin during late blastula and early gastrula stages, but the molecular basis for this distinction is not fully understood [2], [62], [84], [85], [86]. Genes downstream of *ndr1*, like *ntl*, are also induced differentially in the axis versus the ventrolateral margin [62], [85]. Axial *ntl* is required for notochord formation, whereas non-axial *ntl* expression is required to establish ventroposterior mesoderm [87], [88]. Analyses of reporter gene constructs driven by different *ntl* enhancers indicate that, aside from Nodal signaling, both Wnt8a and BMP signaling contribute significantly to the ventrolateral expression of *ntl* [89]. In *ints6* mutant embryos, loss of the non-axial expression domain of *ntl* is likely due to reduced ventrolateral BMP and Wnt8a signaling, thus leading to loss of non-axial mesoderm with a concomitant expansion of axial mesoderm.

### *ints6* mutants are deficient in ventrolateral Wnt8a signaling

Vox, Vent, and Ved are three critical repressors of organizer gene expression that are required to maintain the integrity of non-axial mesoderm. Initial induction of *vox* and zygotic *ved* expression is mediated through *runx2bt2*, a maternally provided splice isoform of *runx2*. Depletion of Runx2bt2 leads to a notable absence of *ved* expression at a late blastula stage, while *vox* and *vent* are expressed [17]. These defects are distinct from that of *ints6* mutants, which display nearly absent *vox* and *ved* expression by early gastrulation.

The Wnt8a pathway is a critical negative regulator of organizer gene expression in ventrolateral non-axial mesoderm operating through Vox, Vent, and Ved [7], [10], [18]. *wnt8a* is induced in a reduced domain in *ints6* mutant embryos but is not maintained by an early gastrula stage (Figure 4B). *ints6* mutants exhibit reduced expression of *vox* and *ved* by the early gastrula stage, a time when these transcriptional repressors rely on Wnt8a for their expression. By the early gastrula stage, organizer gene derepression is evident in *ints6* mutants (Figure 3B). Loss of Wnt8a signaling may cause organizer gene expression to expand, or alternatively the expanded expression of organizer genes in *ints6* mutants may cause the loss of Wnt8a signaling.

Simultaneous loss of both zebrafish β-catenin proteins causes *gsc* and *chd* expression to expand around the embryonic margin, similarly to *ints6* mutants [90]. β-catenin 1 and 2 function redundantly to transduce zygotic Wnt8a signaling, which has been proposed to suppress the *ndr1*-mediated induction of *chd* and *gsc* in the ventrolateral margin [91]. Loss of *wnt8a* expression in *ints6* mutant embryos could similarly cause *chd* and *gsc* to expand around the ventrolateral margin. Our results suggest that Wnt8a signal transduction mechanisms are intact in *ints6* mutants, given that *chd* MO-mediated rescue of *p18ahub* depends on Wnt8a signaling.

### The dorsalization of *ints6* mutants

Our results indicate that *ints6* mutants are not mechanistically defective in BMP signaling. DV patterning of *ints6* mutants can be restored to nearly WT by depleting the BMP antagonist Chd alone, or along with Noggin1 and Follistatin-like 2b. Induction of *bmp2b* expression is normal in *ints6* mutants and loss of *bmp2b and bmp4* expression by early gastrulation likely is the result of expanded organizer gene expression, including *chd* expression, or loss of Wnt8 signaling.

Maternal-zygotic *pou5f3* (MZ*pou5f3*) mutants, like *ints6* mutants, display expanded organizer gene expression at late blastula stages and are severely dorsalized. In contrast to *ints6* deficient embryos, MZ*pou5f3* mutants fail to initiate *bmp2b* and *bmp4* expression, and show greatly reduced *bmp7* expression [20]. In MZ*pou5f3* mutants, expansion of organizer gene expression is likely due to loss of repressors that rely on Pou5f3 for their expression [24]. Similarly to *ints6* mutants, *pou5f3* mutants exhibit aberrant morphogenesis and also display a delay in the completion of epiboly, [22], [92].

However, Pou5f3 has additional functions during gastrulation compared to Ints6. MZ*pou5f3* mutants fail to form endoderm and exhibit reduced *sox17* expression in dorsal forerunner cells [20], [24], in contrast to the expanded populations of *sox17*-expressing forerunner cells observed in *p18ahub* embryos. Endodermal *sox17* expression was observed in *ints6* mutant embryos, although it was restricted more animally than in WT (Figure 5L). We examined the expression of *pou5f3* in *p18ahub* embryos at a late blastula stage and observed no differences from WT (not shown). Thus, *pou5f3* expression does not rely on *ints6*. It is possible that *ints6* and *pou5f3* cooperate in DV patterning and in morphogenesis of the early embryo.

The definitive placement of *ints6* within a genetic pathway as well as the determination of its precise molecular functions will require biochemical characterization of the Ints6 protein. Ints6 may mediate DV patterning by supporting the expression or function of the Wnt8a pathway or specifically modulating the axial versus non-axial response to Nodal. Importantly, our molecular genetic approach has revealed a novel function for Ints6 in vertebrate embryonic patterning. Future studies will reveal its function within the complex gene regulatory network that restricts the organizer to dorsal regions and modulates axial versus non-axial tissues of the vertebrate body plan.

## Materials and Methods

### Ethics statement

The animal work performed in this study was approved by the Institutional Review Board of the University of Pennsylvania.

### Zebrafish strains

*p18ahub* was isolated in an ENU-induced mutagenesis genetic screen for maternal-effect mutations utilizing a hybrid AB/TU genetic background for mapping purposes, similar to that described previously [41], [42]. The *p18ahub* mutation was found to be on the TU chromosome. Thus the line was outcrossed to AB to maintain it. Mutant females from both the original hybrid line as well as outcrossed lines were used. Embryos for *in situ* hybridization and injection experiments were derived from crosses of *p18ahub* mutant females to TL males. Due to delay in morphogenesis, the stage of *p18ahub* embryos is often based on the stage of corresponding age-matched WT reference embryos. Other criteria for embryo staging are described in the text.

### *In situ* hybridization

Antisense RNA probes were synthesized from linearized plasmid templates and transcribed using either SP6 or T7 polymerases (Promega) and Roche digoxygenenin-labeled NTP mix (11277073910). To examine the expression of *ints6* and *ddx26b*, full-length open reading frames were amplified using the following primers and cloned into pDONR221 and ultimately pCSDest using the Gateway system (Invitrogen). Note: coding sequences or complements are shown; attB1 and attB2 sites are omitted.

*ints6* gateway For GTCCATGTAGAAGGGGCGAATATCAAC

*ints6* gateway Rev GTGCTCGAGTCCTTCAAGTAGGGCAG

*ddx26b* gateway For GAGATAGGTCATATGCCGATTGTAGC

*ddx26b* gateway Rev TGAGATGTGACTTGCCACTATCTGC

The hybridization procedure was essentially as described at the Zebrafish Information Network (see http://zfin.org/zf_info/zfbook/chapt9/9.82.html), except Roche BM Purple alkaline phosphatase substrate (11442074001) was used and terminated by washing embryos briefly in PBS followed by overnight fixation in 4% paraformaldehyde/PBS at 4°C. Stained embryos were stored in methanol at −20°C and cleared in a 2∶1 mixture of benzyl benzoate∶benzyl alcohol for photography. Embryos were mounted in Canada Balsam and oriented under glass coverslips for imaging on either a Zeiss Axioscope microscope fitted with a ProgRes CF CCD camera (Jenoptik) or Leica MZ12-5 dissecting microscope fitted with a Photometrics CoolSnap CF CCD camera (Roper Scientific). *In situ* hybridization was performed 2 to 4 times for each probe independently on groups of mutant and age- or stage-matched WT control embryos, as indicated in the text, typically obtained from crosses on the same day.

### Immunostaining for β-catenin

Embryos were fixed overnight at 4°C in 4% paraformaldehye/PBS and washed several times in PBS. Embryos were then dechorionated and placed in 100% methanol overnight or for long-term storage at −20°C. Embryos were rehydrated first in 50% MeOH for 10 minutes and then 3 times for 10 minutes in100% PBST (PBS with 0.5% Triton X-100). Embryos were blocked in Blocking Solution (10% fetal bovine serum/PBST) for one hour before receiving a 1∶500 dilution of rabbit anti-β-catenin antibody (Sigma C2206) in Blocking Solution and incubating at 4°C overnight. The next day embryos were washed 3 times for 10 minutes with 1 ml of Blocking Solution at room temperature before incubation in a 1∶500 dilution of HRP-conjugated goat-anti-rabbit antibody (Sigma A9169) in Blocking Solution for at least two hours at room temperature. Embryos were then washed 4 times in 1 ml PBST and developed in 1 ml of a 1∶3 dilution of a 15 ml solution of 50 mM Tris-Cl pH 7.5, 100 mM NaCl, 1 DAB (3,3′ diaminobenzidine) tablet (Sigma D5905), and 15 µl freshly added 30% H_2_O_2_. Staining was monitored visually under a dissecting microscope and terminated via washing in 50 mM Tris-Cl pH 7.5, 100 mM NaCl. Stained embryos were placed in 100% methanol for storage at −20°C and photographed as described above for *in situ* hybridized embryos.

### Microinjection of embryos

mRNA or morpholinos (MOs) were injected into the yolk cell of 1- to 2-cell stage embryos that were subsequently scored at 24 hours post fertilization (hpf) for phenotypes. *In vitro* transcribed mRNAs were generated from linearized plasmid DNA templates using the mMessage mMachine kit (Ambion). mRNAs were diluted to appropriate working concentrations in 100 mM KCl/10% Phenol Red just prior to injection. Templates for *ints6* (WT or mutant) and *ddx26b* mRNAs were the same as those used to make *in situ* riboprobes described above. Other templates included: *lft1*-pCS2 [64], *sqt*-pCS2 [93], and FLAG-*bmp2b* [94]. MOs were obtained from GeneTools and prepared as stocks in water according to the manufacturers recommendations. Prior to injection, MOs were diluted to working concentrations in 1× Danieau's Solution (58 mM NaCl, 0.7 mM KCl, 0.4 mM MgSO_4_, 0.6 mM Ca(NO_3_)_2_, 5 mM Hepes pH 7.1–7.3). The sequences of the *chordin*, *noggin1*, and *follistatin-like2b* translation-blocking MOs are reported elsewhere [54]. We employed anti-*chd* MO at 0.82 ng/µl, anti-*nog* MO at 4.22 ng/µl, and anti-*fstl2b* MO at 8.45 ng/µl and typically injected 1 nl. To target processing of *wnt8a* transcripts we employed the splice-blocking MOs *orf1 E1i1* and *orf2 E4i4*, as described [60].

### Positional cloning of *p18ahub*

Our fine mapping strategy has been described elsewhere [41], [95]. Heterozygous *p18ahub* females and homozygous *p18ahub* males from the original AB/TU hybrid strain were crossed to obtain females recombinant between SSLP markers flanking the mutation. The initial interval placed *p18ahub* between z34824 and z6845. In total we examined 1148 meioses, narrowing the interval to approximately 1.35 Mb between z7120 and an SSLP marker that we generated against BAC CR545476 (forward primer – AGATGTAACTCATCCACTGTCATACACC, reverse primer – AACCGTTGAGAGGTTTCTAGCTAGTAC). We then proceeded to sequence all genes within the interval for which a cDNA was reported. We made oligo-dT-primed cDNA from the TU wild-type and the *p18ahub* mutant strains from total ovary RNA extracted using Trizol Reagent (Invitrogen 15596-018) according to the manufacturer's instructions, and PCR amplified the coding regions of candidate genes. PCR products were purified (Qiagen PCR clean-up kit) and both strands were sequenced. The *ints6* ORF was sequenced using the primers described above (without att sites) (GenBank Accession number KF700696) and a T to A transition at nucleotide 1124 converting valine 375 to an aspartate was discovered in *p18ahub*-derived ovary cDNA. No mutation was found in genomic DNA derived from the G0 mutagenized male, consistent with the point mutation resulting from ENU-induced mutagenesis.

### Time-lapse imaging

Embryos were mounted in 0.3% low-melt agarose in E3 media and photographed with a Leica MZ12.5 microscope and Micropublisher 5.0 RTV Non-Cooled camera at 5 minute intervals from mid blastula through early somitogenesis stages at room temperature under constant illumination. Images were combined into movies using ImageJ.

## Supporting Information

Movie S1 **Time-lapse movie of** ***p18ahub*** **mutant and wild-type embryos.** Three wild-type and five *p18ahub* mutant embryos from blastula to mid-somitogenesis stages.
(AVI)

Movie S2 **Time-lapse movie of** ***p18ahub*** **mutant embryos.** Six mutant embryos are shown beginning at a mid blastula stage.
(AVI)
